# Nucleosome assembly protein 1-like 5 alleviates Alzheimer’s disease-like pathological characteristics in a cell model

**DOI:** 10.3389/fnmol.2022.1034766

**Published:** 2022-12-08

**Authors:** Bingyan Wang, Weiying Liu, Fengxian Sun

**Affiliations:** ^1^Department of Anesthesiology, The Second Hospital of Tianjin Medical University, Tianjin, China; ^2^Department of Pathogen Biology, School of Basic Medicine, Tianjin Medical University, Tianjin, China; ^3^Department of Physiology and Pathophysiology, School of Basic Medicine, Tianjin Medical University, Tianjin, China

**Keywords:** NAP1L5, Alzheimer’s disease, APP metabolism, neuroinflammation, Tau phosphorylation, GSK3B, Wnt/β-catenin signaling pathway

## Abstract

Alzheimer’s disease (AD) remains one of the most common dementias of neurodegenerative disease-related diseases. Nucleosome assembly protein 1-like 5 (NAP1L5) belongs to the NAP1L protein family, which acts as a histone chaperone. However, the function and mechanism of NAP1L5 in AD are still unclear. Bioinformatics analysis, RT-qPCR, and Western blotting results showed that NAP1L5 was downregulated in the brain tissues of AD patients and a mouse cell model of AD. NAP1L5 overexpression alleviated (Amyloid-β precursor protein) APP metabolism and Tau phosphorylation. We further demonstrated that NAP1L5 regulated the AD-like pathological characteristics through the GSK3B/Wnt/β-Catenin signaling pathway. Moreover, we showed that the Wnt/β-Catenin signaling pathway, regulated by NAP1L5, was mediated by AQP1-mediated mechanism in N2a-APP695sw cell. In sum, these results suggested that NAP1L5 overexpression has neuroprotective effects and might act as potential biomarker and target for the diagnosis and treatment of AD.

## Introduction

Alzheimer’s disease (AD) remains to be the most common form of neurodegenerative disease-related dementia and is observed in millions of elders worldwide (Tahami Monfared et al., 2022). The hallmarks of AD pathology include progressive memory loss and neuropathology, which is mainly caused by extracellular amyloid-beta (Aβ) plaques, neurofibrillary tangles, neuronal loss, and neuroinflammatory responses (Guo et al., 2020). Despite decades of research, the molecular mechanism of AD pathogenesis is unclear, therefore, no effective treatments, especially drugs, have been shown to slow the progression of AD (Mangialasche et al., 2010; Briggs et al., 2016; Haass and Selkoe, 2022; Panza and Lozupone, 2022; Slomski, 2022; Topal et al., 2022; Vandendriessche et al., 2022). Therefore, it is urgent to explore alternative targets to uncover the pathogenesis as well as for AD diagnosis and treatment.

Nucleosome assembly protein 1-like (NAP1L) family members act as histone chaperones and are involved in nucleosome assembly and disassembly, histone transport, and histone eviction with different histone variants (Loyola and Almouzni, 2004; Park and Luger, 2006; Okuwaki et al., 2010). In humans and mice, the NAP1L family contains at least five members, namely NAP1L1–5, and three of them (NAP1L2, NAP1L3, and NAP1L5) have been demonstrated to express with only one copy of a single-exon gene in the neuronal system (Rougeulle and Avner, 1996; Watanabe et al., 1996; Shen et al., 2001; Smith et al., 2003). The other two NAP1-like proteins, identified as the histone chaperones with similar role, are shown to be ubiquitously expressed and do not necessarily have overlapping functions (Simon et al., 1994; Rodriguez et al., 1997, 2000; Okuwaki et al., 2010). Until recently, the functions of the NAP1L5 were largely unknown. NAP1L5, first identified in the human liver malignancy as an imprinted gene, was also found to be hypomethylated in congenital heart diseases (Harada et al., 2002; Smith et al., 2003; Chang et al., 2021). Recently, Wang et al. showed that NAP1L5 promoted nucleolar hypertrophy during cardiomyocyte hypertrophy by regulating translation activation (Guo et al., 2021). However, the role of NAP1L5 in AD remains largely unknown.

Herein, we first showed that NAP1L5 expression was significantly downregulated in the brain tissues of AD patients. To rapidly determine the role of NAP1L5 in APP metabolism and Tau phosphorylation, a mouse AD model cell was used in this study. Further studies revealed that NAP1L5 regulated the molecular features of AD through the GSK3B/Wnt/β-Catenin signaling pathway and was mediated by AQP1. Our findings first explored a novel role of NAP1L5 in the etiology of AD and provided a potential target to diagnose and treat AD.

## Materials and methods

### Animals

Mice, housed at 20–22°C in specific pathogen free room, were had *ad libitum* access to food and water, on a 12-h light–12-h dark cycle. The C57BL/6 J female wild-type (WT, 6 months) mice were purchased from the Beijing HFK Bioscience Co., LET. APP/PS1 double transgenic mice (6 months) were purchased from Junke biological Co., LTD (Nanjing, China, strain name, B6/J-Tg (APPswe, PSEN1dE9); stock number, 406). All studies were approved by the Animal Care and Use Committee of Tianjin Medical University.

### Data acquisition and bioinformatics analysis

The Chromatin Immunoprecipitation (CHIP) data was downloaded from the Gene Expression Omnibus (GEO) database, and the platform file was also downloaded to convert the probe matrix into the Gene Symbol matrix. The “Limma” package in R was used to perform differential expression analysis, and the filtering conditions were fold change > 1.5 and value of *p* < 0.05. Differential expression was shown using a heat map using the “PheatMap” package in R. In the heat map, red represents genes with a high expression in the sample, and blue represents genes with a low expression in the sample. The “GGploT2” package in R was used for volcano mapping of the differential genes. In the volcano diagram, red represents upregulated genes and green represents downregulated genes. The “GGPubR” package in R was used to visualize NAP1L5 and draw boxplots.

### Cell culture and cell transfection

Neuro-2a (N2a) and HEK-293 T cells were purchased from American Tissue Culture Collection (ATCC, United States). The N2a-APP695sw cell line that stably expresses the human APP-695 Swedish mutation (K595N/M596L) was established in our laboratory previously (Sun et al., 2020). The N2a, HEK-293 T, and N2a-APP695sw cells were cultured in Dulbecco’s modified eagle medium (DMEM, Gibco, Carlsbad, CA, United States) containing 10% fetal bovine serum (FBS; Gibco, Carlsbad, CA, United States) and 1% penicillin–streptomycin in 5% CO_2_ and 37°C.

Cells were transfected with Entranster^™^-H4000 (Engreen, China) according to the manufacturer’s protocols. pcDNA3 (Vec-NC) and pcDNA3-AQP1 (AQP1) were obtained from GenePharma Co., Ltd. (Shanghai, China).

### Lentiviral vector construction

To construct the NAP1L5-overexpressing vector, the mouse DNA sequence encoding NAP1L5 (NM_021432.2) was synthesized and cloned into the lentiviral vector pLVX-IRES-Puro to generate pLVX-NAP1L5, which was commissioned by General Biology (Anhui) Co., LTD. For the construction of the NAP1L5-knockdown lentiviral vector pLVX-shNAP1L5, sh-NAP1L5 sense: 5′-ccggt*GCCGAGGACGAGGTAATGGAA* **TTCAAGAGA**TTCCATTACCTCGTCCTCGGCTTTTTTg-3′ and sh-NAP1L5 antisense: 5′-aattcGCCGAGGACGAGGTAATGGAA**TTCAAGAGA**TTCCATTACCTCGTCCTCGGCTTTTTTa-3′ (the letters in italics refer to the targeted sequences of *NAP1L5* gene and the letters in bold refer to the loop sequence) were annealed in the DNA annealing buffer using PCR, and the reaction conditions were set as follows: denaturation at 95°C for 2 min and slow cooling to 25°C. The cooling process took at least 45 min. Then, the annealing products were cloned into a retrovirus pLVX-shRNA1 containing the RNA polymerase III promoter between *BamH*I and *EcoR*I restriction sites. The virus packaging system is a three-plasmid system consisting of the target vector (pLVX-NAP1L5 or pLVX-shNAP1L5) and the packaging vectors psPAX2 and pMD2.G, which contain the necessary elements for virus packaging. To construct the recombinant lentivirus, the target vector and packaging vector were transfected into HEK293T cells using the polyethyleneimine (PEI) transfection reagent. The cell supernatant rich in lentiviral particles was collected at 48 and 72 h after transfection. The lentiviral particles were concentrated using ultrafiltration using the Millipore Amicon Ultra-15,100 kDa to obtain high-titer lentiviral concentrate. Finally, the lentiviral titer was determined using the titer dilution assay.

### Real-time quantitative PCR (RT-qPCR)

Total RNA was extracted using the Trizol method, and single-stranded cDNA was synthesized using the reverse transcription kit (PrimeScript^™^ RT reagent Kit with gDNA Eraser; TaKaRa, China). A 20 μl PCR reaction system was prepared as follows: 2 × SYBR Green qPCR Mix (TaKaRa, China) 10 μl, 0.5 μl of the forward and reverse primers (10 μM) each, 1.0 μl of cDNA template, and 8 μl of sterile double distilled water. The PCR reaction conditions were as follows: pre-denaturation at 95°C for 2 min, denaturation at 95°C for 5 s; annealing at 60°C for 30 s, and extension at 72°C for 30 s, 40 cycles. The relative expression of *NAP1L5* mRNA of each group (2^–ΔΔCT^) was calculated using the comparative cycle-threshold (CT) method, and β-actin was used as the internal reference. The PCR primers were synthesized by General Biology (Anhui) Co., Ltd. The RT-qPCR primer sequences are shown in Supplementary Table S1.

### Western blotting

To extract cell proteins, the cells were first washed twice with pre-cooled 1 × phosphate buffer saline (PBS), then Radio-Immunoprecipitation Assay (RIPA) lysate containing protease inhibitors and phosphatase inhibitors was added, and the cells were lysed at 4°C for 30 min, and further lysed using ultrasound. The protein concentration was determined using the Bicinchoninic Acid (BCA) method. All samples were adjusted to the same concentration, 5 × sodium dodecyl sulfate (SDS) loading buffer was added according to the ratio of 1:4 (*v*/*v*) to the protein sample volume, and the samples were denatured at 100°C for 10 min. First, 8–20% SDS-PAGE gels were selected according to the different molecular weights of the target proteins, and then the proteins were transferred to a polyvinylidene fluoride (PVDF) membrane. The PVDF membranes were cut based on the size of the protein marker, and the membrane was blocked using the blocking solution containing 5% (*m*/*v*) skimmed milk powder at room temperature for 1 h. Then, the PVDF membranes were washed using 1 × Tris-buffered saline with Tween-20 (TBST) twice and incubated in the primary antibody dilution solution at 4°C overnight. The concentration of the primary antibodies was shown in Supplementary Table S2. The next day, the PVDF membranes were washed using 1 × TBST, goat anti-rabbit secondary antibodies (1:5,000) were added and the membranes were incubated at room temperature for 2 h, and then the PVDF membranes were washed again using 1 × TBST. The electrochemiluminescence (ECL) method was used for visualization and Image J image processing software was used for analysis.

### Immunofluorescence assay

Paraffin-embedded mouse brain coronal sections (10 μm) were dewaxed and hydrated, followed by antigenic repair and elimination of endogenous peroxidase activity. 5% BSA was added to incubate at 37°C for 30 min. Then, primary antibodies were added and incubated overnight at 4°C. The concentration of the primary antibodies was shown in Supplementary Table S3. Goat Anti-Mouse IgG H&L (Alexa Fluor^®^ 647, 1:100, ab150115, Abcam, United States) and Goat Anti-Rabbit IgG H&L (Alexa Fluor^®^ 488, 1:100, ab150077, Abcam, United States) was added and incubated for 1 h at room temperature without light. DAPI was used to redye the nucleus and the images were acquired using a Zeiss laser confocal microscope.

### TOP/FOP flash luciferase reporter assay

The TOP/FOP flash luciferase reporter assay was performed according to a previously reported method (Yao et al., 2022).

### Statistical analysis

Data are expressed as mean ± standard deviation (mean ± SD). All experiments were repeated three times. SPSS 22.0 and GraphPad Prism 8 were used for data analysis. The student’s independent t-test was used for comparison between the two groups. A *p* < 0.05 was considered statistically significant.

## Results

### NAP1L5 was dysregulated in AD

**Figure 1:**
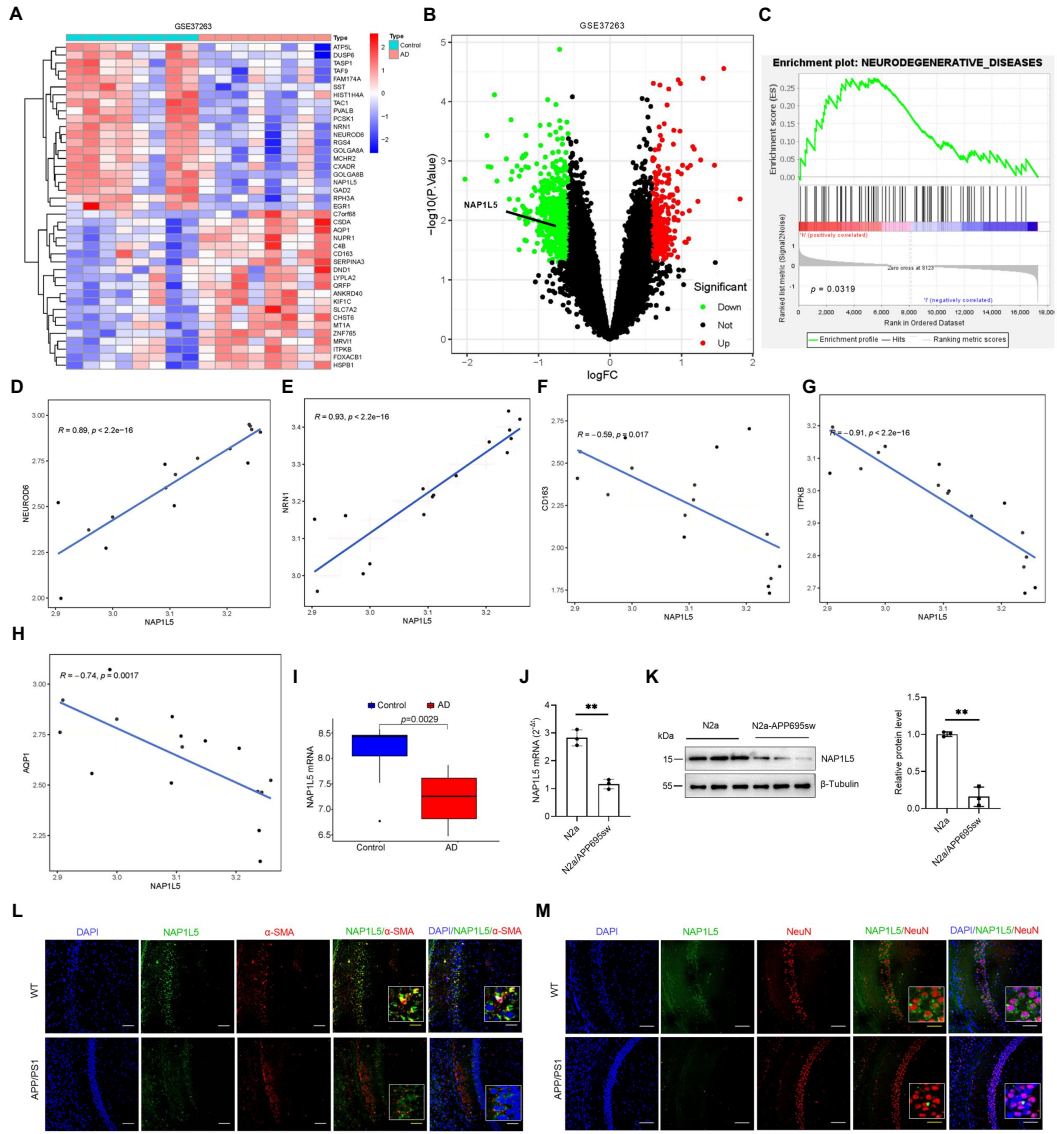
NAP1L5 was downregulated in Alzheimer’s disease (AD) patients and the AD cell model. **(A)** A heatmap showing the differentially expressed genes in the brain tissues of AD patients and controls according to the GEO (GSE37263) dataset. **(B)** Volcano plots showed that NAP1L5 was significantly differentially expressed. **(C)** GSEA analysis showed that NAP1L5 expression was significantly correlated with neurodegenerative diseases in the GSE37263 datasets. **(D–H)** The NAP1L5 expression was significantly correlated with NEUROD6, NRN1, CD163, ITPKB, and AQP1. **(I)** *NAP1L5* mRNA level was analyzed in the brain tissues of AD patients and controls from GSE37263. *NAP1L5* mRNA **(J)** and protein **(K)** levels were detected in the cell model of AD. **(L,M)** Relationship of NAP1L5 with alpha-smooth muscle actin (α-SMA) fraction and neuron. **(L)** Representative immunofluorescence images of hippocampus CA1 showing NAP1L5 (green) merged with α-SMA (red). Scale bars, 100 μm. **(M)** Representative immunofluorescence images of hippocampus CA1 showing NAP1L5 (green) merged with neuron (NeuN, red). Scale bars, 100 μm. Data are present as means ± SD, *n* = 3. ^∗^*p* < 0.05. Student’s test.

Transcriptome sequencing has provided a large amount of clinical data for the study of the mechanism underlying AD. Further bioinformatic analysis of these data can help identify novel candidate pathogenic genes. In the current study, AD patient data from the Gene Chip Public Database (GEO) and GSE37263 microarray data submitted by Tan MG and Lai MK (Tan et al., 2010) was obtained and used for reanalysis using bioinformatic tools. According to the analysis results, NAP1L5 was observed to be downregulated in the brain tissues of AD patients and might regulate the pathological process of AD (Figures 1A,B). To further predict the potential role in AD regulated by NAP1L5, GSEA results from GSE37263 datasets showed that neurodegenerative diseases, the crucial diseases of the nervous system involving Parkinson’s disease and AD, were strongly correlated with the expression level of NAP1L5 (Figure 1C). Among these genes of neurodegenerative diseases, we observed that the NAP1L5 expression was significantly positively correlated with NEUROD6 and NRN1, whereas correlated with negatively CD163, ITPKB, and AQP1 (Figures 1D–H). Consistent with our predictions that NAP1L5 was downregulated in the brain tissues of AD patients (Figure 1I), *NAP1L5* mRNA levels further verified to be reduced in N2a-APP695sw (Figure 1J), a previously verified cell model of AD (Li et al., 2015; Zhang et al., 2017a,b; Sun et al., 2020; Yao et al., 2022). Additionally, the protein level of NAP1L5 was decreased in N2a-APP695sw compared to N2a cells (Figure 1K). Moreover, we detected the expression of NAP1L5 in the brain regions and cell types to investigate the relationship of NAP1L5 with alpha-smooth muscle actin (α-SMA) fraction and neuron. Immunofluorescence results showed that the expression of NAP1L5 was markedly reduced in the hippocampus CA1 region of APP/PS1 mouse (Figures 1L,M). Interestingly, we observed that the NAP1L5 protein was distributed in cytosol rather than in nucleus, and was partly co-localized with α-SMA or expressed in the cytosol of neuron (Figures 1L,M). Similar to previous reports, NAP1L5, formerly known as DRLM, did not have a nuclear localization signal, distributed in cytoplasm (Harada et al., 2002). Together, the results suggested that NAP1L5 might play a certain role in regulating AD etiology figure.

### NAP1L5 regulated APP processing

**Figure 2:**
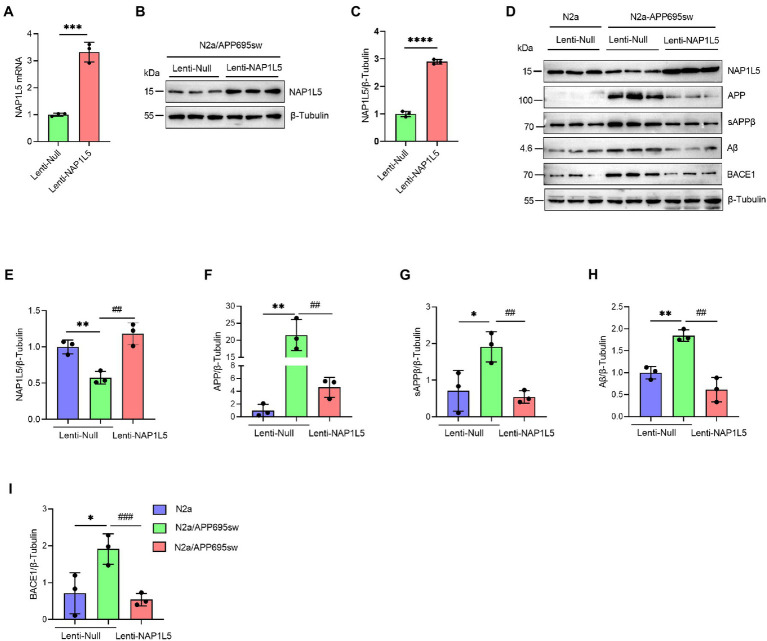
NAP1L5 overexpression inhibited APP metabolism in N2a-APP695sw cells. **(A–C)** N2a-APP695sw cells were stably infected with lenti-Null and lenti-NAP1L5, and the mRNA and protein levels of *NAP1L5* were detected using RT-qPCR and Western blotting, respectively. Data are presented as mean ± SD, *n* = 3. ^∗∗∗∗^*p* < 0.0001; Student’s *t*-test. **(D–I)** N2a or N2a-APP695sw cells were stably infected lenti-Null or lenti-NAP1L5. APP metabolism-related protein levels were detected using Western blotting **(D)**, and then quantified **(E–I)**. Data are presented as mean ± SD, *n* = 3. ^∗^*p* < 0.05; ^∗∗^*p* < 0.01; ^∗∗∗^*p* < 0.001; ^∗∗∗∗^*p* < 0.0001 for N2a-APP695sw (lenti-Null) versus N2a (lenti-Null). ^##^*p* < 0.01; ^###^*p* < 0.001; ^####^*p* < 0.0001 for N2a-APP695sw (lenti-NAP1L5) versus N2a-APP695sw (lenti-Null). The student’s *t*-test was used.

To confirm the role of NAP1L5 in APP processing, we determined the mRNA and protein levels of NAP1L5 in lentivirus-mediated NAP1L5-overexpressing N2a-APP695sw cells. The mRNA and protein levels of NAP1L5 significantly increased in NAP1L5-overexpressing N2a-APP695sw cells (Figures 2A–E). Then, we determined whether NAP1L5 overexpression affected APP metabolism. The APP protein level in lenti-Null N2a-APP695sw cells dramatically increased compared to N2a, whereas NAP1L5 overexpression strongly reduced APP metabolism (Figures 2D,F). sAPPβ is an important metabolic intermediate in the first stage of APP amyloid pathway degradation, which eventually leads to the production of toxic Aβ (Delport and Hewer, 2022). The results showed that sAPPβ and Aβ were significantly enhanced in N2a-APP695sw compared to N2a, however, NAP1L5 overexpression decreased the production of sAPPβ and Aβ in N2a-APP695sw cells (Figures 2D–H). Moreover, β-secretase (BACE1) plays an important role in mediating the first stage of APP proteolysis(Cervellati et al., 2021). Our results showed that NAP1L5 overexpression inhibited BACE1 expression (Figures 2D–I), which was consistent with the inhibition of APP metabolism. Together, the results indicated that NAP1L5 played a pivotal role in regulating APP processing.

### NAP1L5 repressed the hyperphosphorylation of Tau

**Figure 3:**
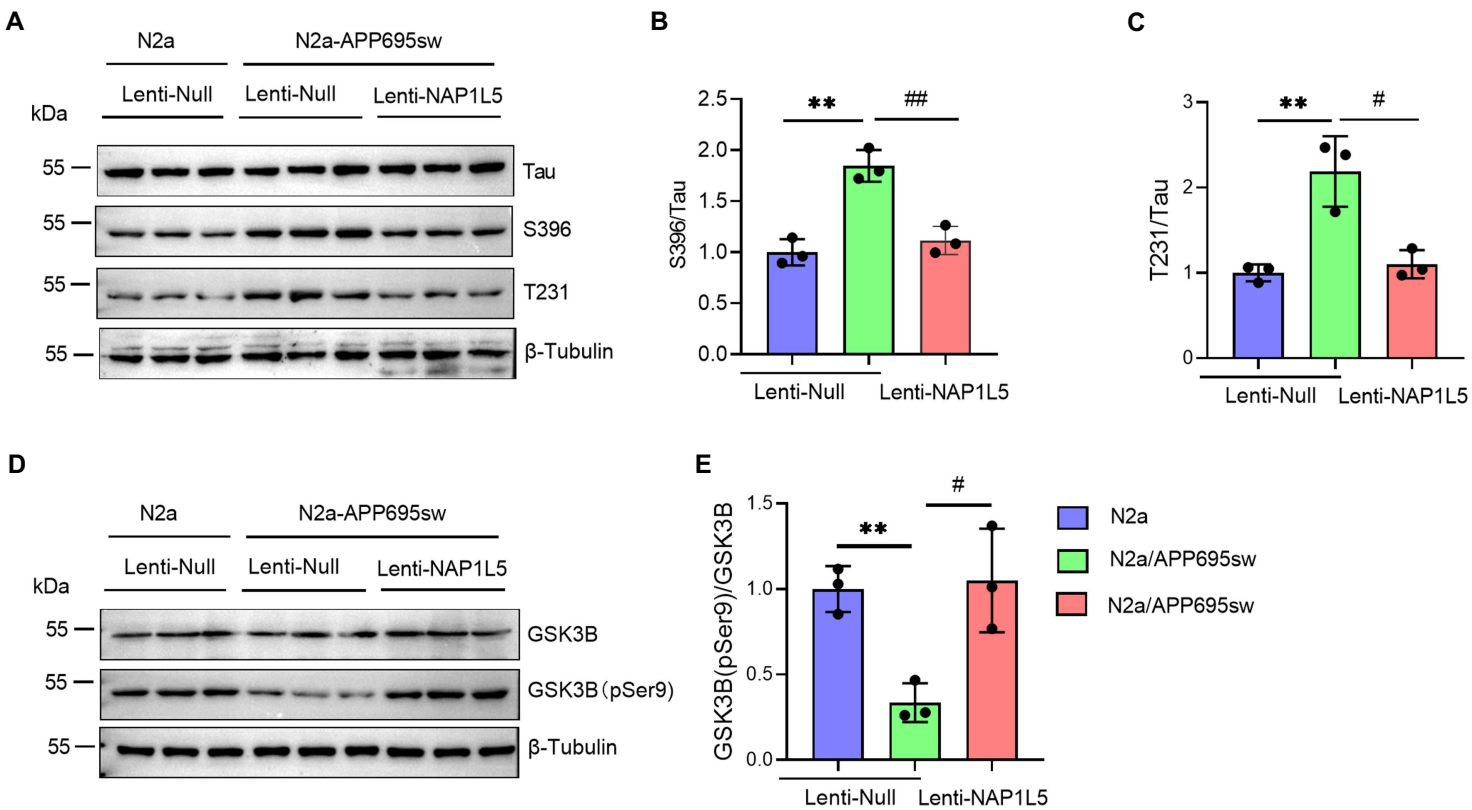
NAP1L5 overexpression abrogated the hyperphosphorylation of Tau but enhanced the phosphorylation of GSK3B at serine 9 in N2a-APP695sw cells. **(A–E)**, N2a and N2a-APP695sw cells were stably infected with lenti-Null and lenti-NAP1L5. The Tau and phosphorylated Tau protein levels were detected using Western blotting **(A)**, and then quantified **(B,C)**. The GSK3B and phosphorylated GSK3B at serine 9 (GSK3B (pSer9)) levels were measured using Western blotting **(D)**, and then quantified **(E)**. Data are presented as mean ± SD, *n* = 3. ^∗∗^*p* < 0.01 for N2a-APP695sw (lenti-Null) versus N2a (lenti-Null). ^#^*p* < 0.05; ^##^*p* < 0.01 for N2a-APP695sw (lenti-NAP1L5) versus N2a-APP695sw (lenti-Null). The student’s *t*-test was used.

Abnormal hyperphosphorylation of Tau, which could cause disruption of microtubules or enhance the misfolding of normal Tau and its co-aggregates into filaments, is one of the hallmarks of AD (Wang et al., 2013). Our results showed that phospho-Tau Ser396 and Thr231 were markedly increased in N2a-APP695sw compared to N2a, whereas NAP1L5 overexpression reversed the increase of phospho-Tau Ser396 and Thr231 in N2a-APP695sw cells (Figures 3A–C). Tau hyperphosphorylation is usually caused by several protein kinases, especially serine/threonine kinases, including the cell-mediated gel contraction (CMGC), calcium/calmodulin-dependent protein kinase (CAMK), and casein kinase 1 (CK1) families, as classified by Manning et al. (2002). Among the CMGC family members, GSK3B has been demonstrated to phosphorylate Tau at most of the known AD sites (Wang et al., 2013). Therefore, we measured the protein levels of total GSK3B and phospho-GSK3B Ser9 in N2a-APP695sw and N2a cells. We observed that the ratio of GSK3B (pSer9) to GSK3B was reduced significantly in lenti-Null N2a-APP695sw cells compared to N2a, whereas these changes were reversed when NAP1L5 was overexpressed in N2a-APP695sw cells (Figures 3D,E).

### NAP1L5 activated the Wnt/β-Catenin signaling pathway

**Figure 4:**
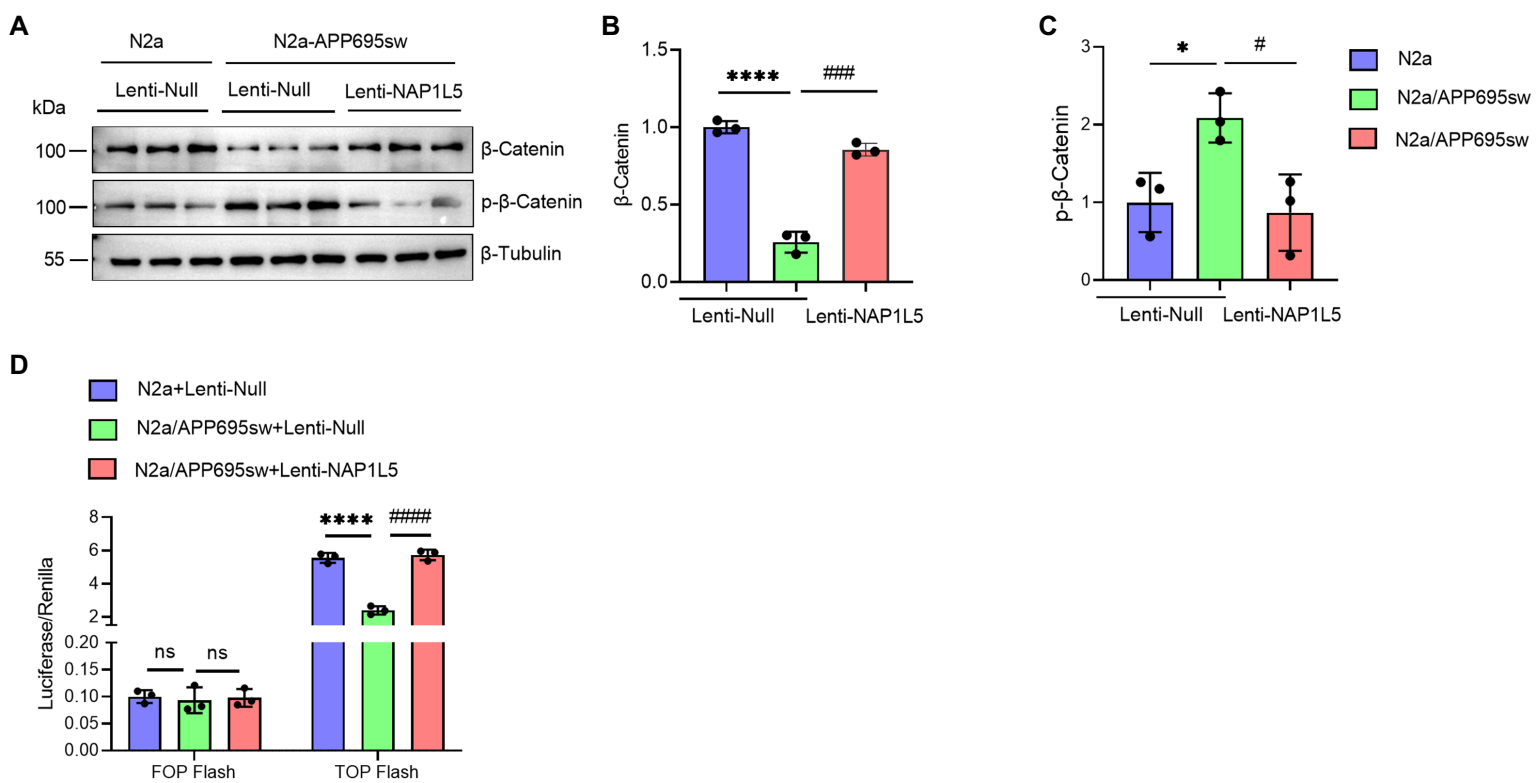
NAP1L5 overexpression activated the Wnt/β-Catenin signaling pathway in N2a-APP695sw cells. **(A–C)**, N2a and N2a-APP695sw cells were stably infected lenti-Null or lenti-NAP1L5. The β-Catenin and phosphorylated β-Catenin levels were detected using Western blotting **(A)**, and then quantified **(B,C)**. **(D)** The activation of the Wnt/β-Catenin signaling pathway was assured using the TOP/FOP flash assay. Data are presented as mean ± SD, *n* = 3. ^∗^*p* < 0.05; ^∗∗∗∗^*p* < 0.0001 for N2a-APP695sw (lenti-Null) versus N2a (lenti-Null). ^#^*p* < 0.05, ^###^*p* < 0.001; ^####^*p* < 0.0001 for N2a-APP695sw (lenti-NAP1L5) versus N2a-APP695sw (lenti-Null); ns, not significant. The student’s *t*-test was used.

Recent studies demonstrated that GSK3B is one of the important mediators of Wnt/β-Catenin signaling pathway (Clevers and Nusse, 2012; Meffre et al., 2014; Nusse and Clevers, 2017). Since NAP1L5 overexpression increased the ratio of GSK3B (pSer9) to GSK3B, we hypothesized that the Wnt/β-Catenin signaling pathway might be activated by NAP1L5. Western blotting results indicated that the β-Catenin protein level was significantly decreased in lenti-Null N2a-APP695sw cells compared to N2a, while phospho-β-Catenin (p-β-Catenin) was significantly increased (Figures 4A–C). We also observed the increase in β-Catenin and the decrease in p-β-Catenin while NAP1L5 was overexpressed in N2a-APP695sw cells (Figures 4A–C). Furthermore, the TOP/FOP flash luciferase reporter assay results showed that the activity of the Wnt/β-Catenin pathway increased in NAP1L5-overexpressing N2a-APP695sw cells (Figure 4D).

### GSK3B inhibitor TDZD-8 alleviated AD-like pathological characteristics

**Figure 5:**
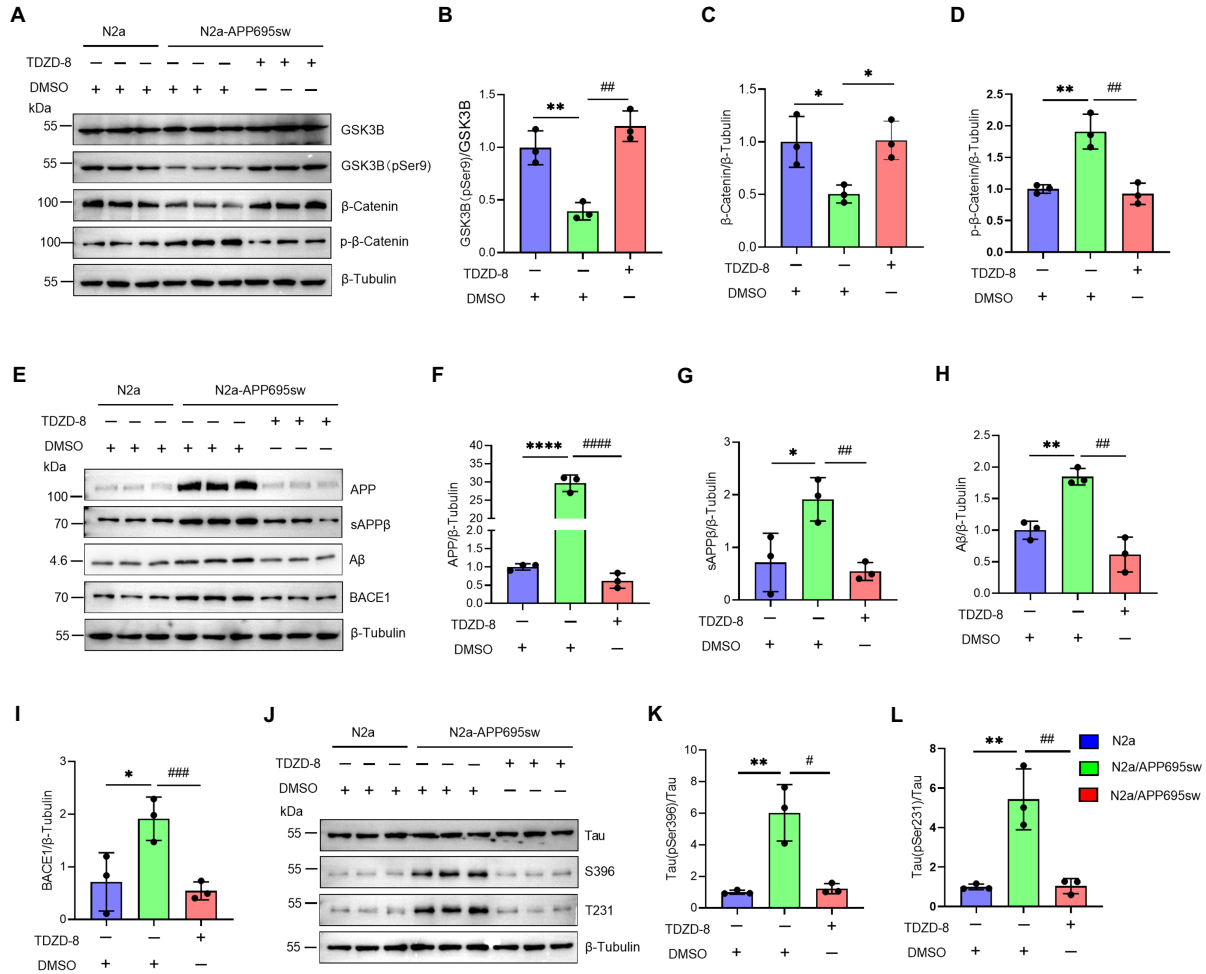
GSK3B inhibitor TDZD-8 promoted APP metabolism and repressed GFAP generation and Tau phosphorylation in N2a-APP695sw cells. **(A–L)**, N2a and N2a-APP695sw cells were treated with DMSO or TDZD-8 (25 μM) for 48 h. The GSK3B, phosphorylated GSK3B at serine 9 [GSK3B (pSer9)], β-Catenin, and phosphorylated β-Catenin were measured using Western blotting **(A)**, and then quantified **(B–D)**. The APP metabolism-related protein levels were measured using Western blotting **(E)**, and then quantified **(F–I)**. The Tau and phosphorylated Tau protein levels were measured using Western blotting **(J)**, and then quantified **(K,L)**. Data are presented as mean ± SD, *n* = 3. ^∗^*p* < 0.05; ^∗∗^*p* < 0.01; ^∗∗∗∗^*p* < 0.0001 for N2a-APP695sw (DMSO) versus N2a (DMSO). ^#^*p* < 0.05; ^##^*p* < 0.01; ^###^*p* < 0.001; ^####^*p* < 0.0001 for N2a-APP695sw (TDZD-8, 25 μM) versus N2a-APP695sw (DMSO). The student’s *t*-test was used.

The previous results confirmed that NAP1L5 regulates the GSK3B/Wnt/β-Catenin axis. Next, we treated the N2a-APP695sw cells with the GSK3B-specific inhibitor TDZD-8 to investigate whether TDZD-8 affected the pathological characteristics of AD. Western blotting results suggested that the ratio of GSK3B (pSer9) to GSK3B reduced in N2a-APP695sw cells compared to N2a, whereas TDZD-8 treatment significantly increased the ratio of GSK3B (pSer9) to GSK3B (Figures 5A,B). Furthermore, the Wnt/β-Catenin signaling pathway could also be activated after TDZD-8 treatment (Figures 5A,C,D). We, then, checked whether the effects of TDZD-8 treatment on the pathological characteristics of AD were consistent with those of NAP1L5 overexpression. The results showed that the effects of TDZD-8 treatment on the pathological characteristics of AD, such as APP metabolism and Tau phosphorylation, were consistent with those of NAP1L5 overexpression (Figures 5E–L).

### NAP1L5 regulated the AD-like pathological characteristics through the GSK3B/Wnt/β-Catenin signaling pathway

**Figure 6:**
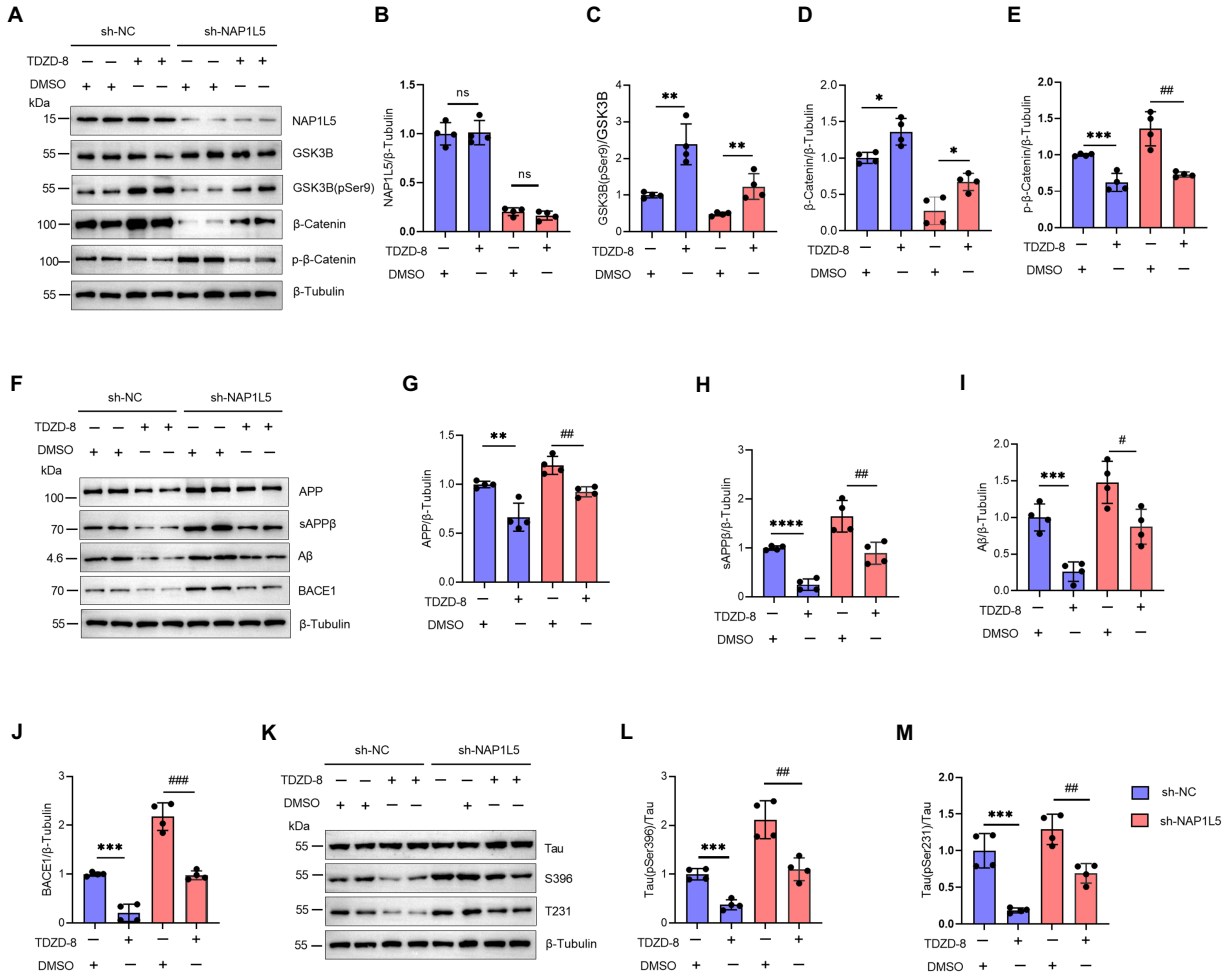
NAP1L5 regulated APP metabolism and Tau phosphorylation through the GSK3B/Wnt/β-Catenin signaling pathway in N2a-APP695sw cells. **(A–M)**, N2a-APP695sw cells, stably transfected with sh-NC or sh-NAP1L5, were treated with DMSO or TDZD-8 (25 μM) for 48 h. The NAP1L5, GSK3B, phosphorylated GSK3B at serine 9 [GSK3B (pSer9)], β-Catenin, and phosphorylated β-Catenin levels were measured using Western blotting **(A)**, and then quantified **(B–E)**. The APP metabolism-related protein levels were measured using Western blotting **(F)**, and then quantified **(G–J)**. The Tau and phosphorylated Tau protein levels were measured using Western blotting **(K)**, and then quantified **(L,M)**. Data are presented as mean ± SD, *n* = 4. ^∗^*p* < 0.05; ^∗∗^*p* < 0.01; ^∗∗∗^*p* < 0.001; ^∗∗∗∗^*p* < 0.0001 for TDZD-8 (25 μM) versus DMSO in N2a-APP695sw cells transfected with sh-NC. ^#^*p* < 0.05; ^##^*p* < 0.01; ^###^*p* < 0.001; ^####^*p* < 0.0001 for TDZD-8 (25 μM) versus DMSO in N2a-APP695sw cells transfected with sh-NAP1L5. ns, not significant; the Student’s *t*-test was used.

In addition, we confirmed that NAP1L5 regulated the AD-like pathological characteristics through GSK3B. We first confirmed that the mRNA and protein levels of NAP1L5 were markedly reduced in NAP1L5-knockdown cells (Supplementary Figures S1A,B; Figures 6A,B). Then, we observed that TDZD-8 treatment increased the ratio of GSK3B (pSer9) to GSK3B in N2a-APP695sw cells stably expressing sh-NC or sh-NAP1L5 vectors (Figures 6A,C). Moreover, the Wnt/β-Catenin signaling pathway was activated after TDZD-8 treatment (Figures 6A,D,E). Then, we checked whether TDZD-8 treatment affected APP metabolism in N2a-APP695sw cells stably expressing sh-NC or sh-NAP1L5 vectors. We observed that the protein levels of APP, sAPPβ, Aβ, and BACE1 in the NAP1L5-knockdown or sh-NC N2a-APP695sw cells treated with TDZD-8 were partially rescued by knockdown of NAP1L5 (Figures 6F–J), indicating that NAP1L5 mediated APP processing through GSK3B. We then checked whether NAP1L5 also regulated Tau phosphorylation through GSK3B. We found that phospho-Tau Ser396 and Thr231 were partially rescued in NAP1L5-knockdown or sh-NC N2a-APP695sw cells treated with TDZD-8 (Figures 6K–M). Taken together, our findings showed that TDZD-8 antagonized AD-like pathological characteristics by preventing NAP1L5 knockdown-mediated toxic effects.

### NAP1L5 reduced AD-like pathological characteristics by downregulation of AQP1

**Figure 7:**
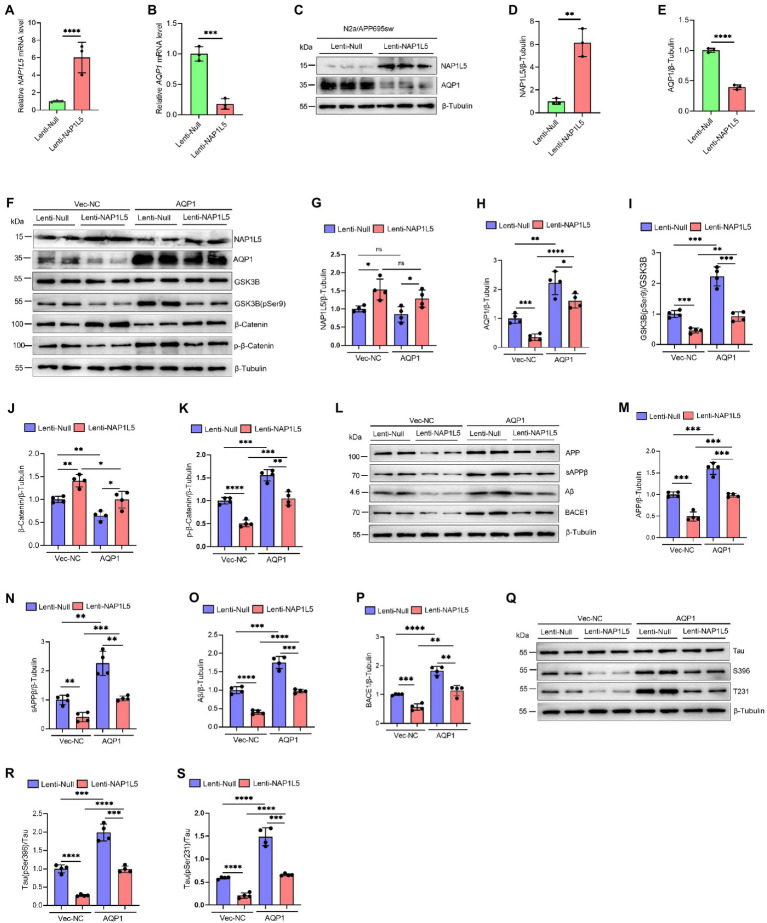
NAP1L5 alleviated AD-like pathological characteristics through AQP1-mediated mechanism in N2a-APP695sw cell. **(A–E)** N2a-APP695sw cells were stably infected Lenti-Null and Lenti-NAP1L5, NAP1L5 and AQP1 mRNAs were determined by RT-qPCR **(A,B)**; the protein levels of NAP1L5 and AQP1 were measured by Western blot assays **(C–E)**. Data are present as means ± SD, *n* = 3. ^∗∗^*p* < 0.01, ^∗∗∗^*p* < 0.001 or ^∗∗∗∗^*p* < 0.0001 by Student’s test. **(F–S)**, N2a-APP695sw cells, stably infected with Lenti-Null and Lenti-NAP1L5, were transfected with the control vector (Vec-NC) or AQP1 overexpressing vector (AQP1) for 48 h. Then, the proteins were extracted and the protein levels of related genes were detected by Western blot assays. **(F–K)** NAP1L5, AQP1, GSK3B, phosphorylation of GSK3B at serine 9 [GSK3B (pSer9)], β-Catenin and phosphorylation of β-Catenin were detected by Western blot assays and quantified. **(L–P)** APP metabolism-related protein levels were detected by Western blot assays and quantified. **(Q–S)** Tau protein and phosphorylation of Tau proteins were detected by Western blot assays and quantified. Data are present as means ± SD, *n* = 4. ^∗^*p* < 0.05, ^∗∗^*p* < 0.01, ^∗∗∗^*p* < 0.001 or ^∗∗∗∗^*p* < 0.0001. Student’s test.

Our previous predictions have shown that NAP1L5 is negatively correlated with the expression level of AQP1, which has been suggested as a key regulator involved in the pathogenesis of AD (Misawa et al., 2008; Ijaz et al., 2021; Park et al., 2021). Noteworthy, AQP1 was also the trigger of Wnt/β-Catenin signaling pathway. Downregulation of AQP1 could ameliorate cognitive function by activating the Wnt/β-Catenin signaling pathway in a mouse model of AD (Yu et al., 2020). Therefore, we wondered whether NAP1L5 regulated the Wnt/β-Catenin signaling pathway through a molecular mechanism mediated by AQP1. We first showed that the mRNA and protein levels of AQP1 were markedly reduced in NAP1L5 overexpressing N2a-APP695sw cells (Figures 7A–E). Next, we showed that overexpression of AQP1 could partly counteract the activation of Wnt/β-Catenin signaling pathway induced by NAP1L5 (Figures 7F–K). These interesting results led us to ask whether AQP1 also involved in regulation of APP metabolism and Tau phosphorylation in NAP1L5 overexpressing N2a-APP695sw cells. In line with our findings from bioinformatics prediction, the process of APP metabolism to produce Aβ was partially restored in NAP1L5 overexpressing N2a-APP695sw cells while AQP1 was co-overexpressed (Figures 7L–P). Similarly, NAP1L5 overexpression could inhibit the hyperphosphorylation of Tau, while co-overexpression of AQP1 partly abolished the inhibition on hyper-phosphorylation of Tau protein (Figures 7Q–S). Collectively, these results suggested that NAP1L5 alleviated AD-like pathological characteristics through AQP1-mediated mechanism in N2a-APP695sw cell.

## Discussion

Increasing evidence indicates that AD is a complex neurodegenerative disease. Although most clinical trials have failed for a multitude of reasons, the understanding of the underlying molecular mechanisms of AD is still in its infancy because of the complexity of its pathophysiology (Asher and Priefer, 2022; Qiu, 2022; Therriault et al., 2022; Uwishema et al., 2022). More specific biomarkers or therapeutic targets that can accelerate the efforts to improve and simplify the areas of differential diagnosis and understand the molecular basis of AD are urgently needed (Koníčková et al., 2022).

As one of the members of the NAP1L protein family, NAP1L5 was identified as an imprinted gene in human liver malignancy and congenital heart diseases and an important regulator of translation activation during cardiomyocyte hypertrophy (Harada et al., 2002; Chang et al., 2021; Guo et al., 2021). However, the role of NAP1L5 in various diseases, including AD, remains largely unknown. Herein, we first investigated the relationship between the expression of NAP1L5 and AD. GSE37263 microarray data reanalysis using bioinformatic tools revealed that NAP1L5 was reduced in the brain tissues of AD patients, indicating that it might play a role in the pathological process of AD. Consistent with these results, NAP1L5 expression was confirmed to be decreased in a cell model of AD. In addition, NAP1L5, upon the results form bioinformatics analysis, was strongly correlated with neurodegenerative diseases. Further analysis suggested that the NAP1L5 expression was significantly correlated with NEUROD6, NRN1, CD163, ITPKB, and AQP1, which have been proven to play important role in AD (Pey et al., 2014; Fowler et al., 2015; Piras et al., 2019; Zhang et al., 2020; Park et al., 2021).The accumulation of Aβ peptides has been demonstrated to be one of the hallmarks of AD that can cause oxidative stress, neuroinflammatory responses, mitochondrial dysfunction, and endosome–lysosome abnormalities, which all lead to cognitive and memory deficits in several Tg AD mice models (Cheignon et al., 2018; Butterfield and Halliwell, 2019; Samaey et al., 2019; Brewer et al., 2020; Huang et al., 2020; Ismail et al., 2020; Marshall et al., 2020). APP can be processed to produce Aβ through the amyloidogenic pathways consisting of a series of cleavage enzymes like β-secretase and γ-secretase complex (Delport and Hewer, 2022). In the current study, our results showed that NAP1L5 overexpression strongly inhibited the protein levels of APP, sAPPβ, and Aβ in N2a-APP695sw cells, indicating that both APP protein level and the amyloid pathway degradation might be inhibited. Moreover, NAP1L5 overexpression significantly reduced the β-secretase BACE1 expression level, which was consistent with the inhibition of APP metabolism. Together, we showed that NAP1L5 played a crucial role in regulating APP expression and APP processing in N2a-APP695sw cells.

The tauopathies, a class of diseases caused by the misfolding of the Tau protein, were also the main pathological characteristics of AD that induced the abnormal hyperphosphorylation of Tau (Wang et al., 2013). In the current study, we observed that NAP1L5 overexpression markedly reduced phospho-Tau Ser396 and Thr231 in N2a-APP695sw cells, indicating that NAP1L5 overexpression could alleviate the tauopathies of AD. We further demonstrated that NAP1L5 also regulated the activity of GSK3B, a protein kinase that can phosphorylate Tau at most of the known AD sites, by phosphorylating GSK3B at Ser9, whose activity also involves in the Wnt/β-Catenin signaling pathway (Clevers and Nusse, 2012; Meffre et al., 2014; Nusse and Clevers, 2017). Our results showed that the Wnt/β-Catenin signaling pathway could be activated by NAP1L5 overexpression. GSK3B inhibitor TDZD-8 exerted a similar effect as NAP1L5 overexpression on the pathological characteristics of AD. We further showed that TDZD-8 antagonized AD-like pathological characteristics by preventing NAP1L5 knockdown-mediated toxic effects, indicating that NAP1L5 regulated AD-like pathological characteristics by regulation of the GSK3B/Wnt/β-Catenin signaling pathway.

To date, there is still a gap between the decreased NAP1L5 and the inhibition of Wnt signaling pathway and no evidence have shown the interaction between NAP1L5 and Wnt pathway. Herein, we found that AQP1, a key regulator or trigger of Wnt/β-Catenin signaling pathway involved in the pathogenesis of AD (Misawa et al., 2008; Ijaz et al., 2021; Park et al., 2021), was negatively correlated with NAP1L5 expression, indicating that AQP1 might involve in NAP1L5-mediated regulation of the Wnt/β-Catenin signaling pathway. As expected, we have shown that NAP1L5 could alleviate AD-like pathological characteristics through AQP1-mediated mechanism in N2a-APP695sw cell.

In summary, our results first showed that NAP1L5 was downregulated in the brain tissues of AD patients as well as in a cell model of AD. The function of NAP1L5 was confirmed by the detection of the key pathological characteristics of AD using cellular experiments. Further investigation of the mechanism of NAP1L5 indicated that NAP1L5 overexpression inhibited the AD-like pathological characteristics through the GSK3B/Wnt/β-Catenin signaling pathway, which could be mediated by AQP1. These findings demonstrated a novel role of NAP1L5 and suggested that NAP1L5 might be a potential target to diagnose and treat AD.

## Data availability statement

The original contributions presented in the study are included in the article/Supplementary material, further inquiries can be directed to the corresponding authors.

## Ethics statement

The animal study was reviewed and approved by Animal Care and Use Committee of Tianjin Medical University.

## Author contributions

WL and FS conceived the study. WL, FS, and BW performed the experiments, designed all figures and wrote the manuscript. All authors contributed to the article and approved the submitted version.

## Fundings

This work was supported by the Research Project of Tianjin Education Commission (2019KJ172).

## Conflict of interest

The authors declare that the research was conducted in the absence of any commercial or financial relationships that could be construed as a potential conflict of interest.

## Publisher’s note

All claims expressed in this article are solely those of the authors and do not necessarily represent those of their affiliated organizations, or those of the publisher, the editors and the reviewers. Any product that may be evaluated in this article, or claim that may be made by its manufacturer, is not guaranteed or endorsed by the publisher.

## References

[ref1] Asher S. Priefer R. (2022). Alzheimer's disease failed clinical trials. Life Sci. 306:120861. doi: 10.1016/j.lfs.2022.120861, PMID: 35932841

[ref2] Brewer G. J. Herrera R. A. Philipp S. Sosna J. Reyes-Ruiz J. M. Glabe C. G. (2020). Age-related Intraneuronal aggregation of amyloid-β in endosomes, mitochondria, Autophagosomes, and lysosomes. J. Alzheimers Dis. 73, 229–246. doi: 10.3233/JAD-190835, PMID: 31771065 PMC7029321

[ref3] Briggs R. Kennelly S. P. O'neill D. (2016). Drug treatments in Alzheimer's disease. Clin. Med. (Lond.) 16, 247–253. doi: 10.7861/clinmedicine.16-3-247, PMID: 27251914 PMC5922703

[ref4] Butterfield D. A. Halliwell B. (2019). Oxidative stress, dysfunctional glucose metabolism and Alzheimer disease. Nat. Rev. Neurosci. 20, 148–160. doi: 10.1038/s41583-019-0132-6, PMID: 30737462 PMC9382875

[ref5] Cervellati C. Valacchi G. Zuliani G. (2021). BACE1 role in Alzheimer's disease and other dementias: from the theory to the practice. Neural Regen. Res. 16, 2407–2408. doi: 10.4103/1673-5374.313041, PMID: 33907020 PMC8374572

[ref6] Chang S. Wang Y. Xin Y. Wang S. Luo Y. Wang L. (2021). DNA methylation abnormalities of imprinted genes in congenital heart disease: a pilot study. BMC Med. Genet. 14:4. doi: 10.1186/s12920-020-00848-0, PMID: 33407475 PMC7789576

[ref7] Cheignon C. Tomas M. Bonnefont-Rousselot D. Faller P. Hureau C. Collin F. (2018). Oxidative stress and the amyloid beta peptide in Alzheimer's disease. Redox Biol. 14, 450–464. doi: 10.1016/j.redox.2017.10.014, PMID: 29080524 PMC5680523

[ref8] Clevers H. Nusse R. (2012). Wnt/β-catenin signaling and disease. Cells 149, 1192–1205. doi: 10.1016/j.cell.2012.05.012 22682243

[ref9] Delport A. Hewer R. (2022). The amyloid precursor protein: a converging point in Alzheimer's disease. Mol. Neurobiol. 59, 4501–4516. doi: 10.1007/s12035-022-02863-x, PMID: 35579846

[ref10] Fowler K. D. Funt J. M. Artyomov M. N. Zeskind B. Kolitz S. E. Towfic F. (2015). Leveraging existing data sets to generate new insights into Alzheimer's disease biology in specific patient subsets. Sci. Rep. 5:14324. doi: 10.1038/srep14324, PMID: 26395074 PMC4585817

[ref11] Guo T. Zhang D. Zeng Y. Huang T. Y. Xu H. Zhao Y. (2020). Molecular and cellular mechanisms underlying the pathogenesis of Alzheimer's disease. Mol. Neurodegener. 15:40. doi: 10.1186/s13024-020-00391-7, PMID: 32677986 PMC7364557

[ref12] Guo N. Zheng D. Sun J. Lv J. Wang S. Fang Y. (2021). NAP1L5 promotes Nucleolar hypertrophy and is required for translation activation during Cardiomyocyte hypertrophy. Front. Cardiovasc. Med. 8:791501. doi: 10.3389/fcvm.2021.791501, PMID: 34977198 PMC8718910

[ref13] Haass C. Selkoe D. (2022). If amyloid drives Alzheimer disease, why have anti-amyloid therapies not yet slowed cognitive decline? PLoS Biol. 20:e3001694. doi: 10.1371/journal.pbio.3001694, PMID: 35862308 PMC9302755

[ref14] Harada H. Nagai H. Ezura Y. Yokota T. Ohsawa I. Yamaguchi K. (2002). Down-regulation of a novel gene, DRLM, in human liver malignancy from 4q22 that encodes a NAP-like protein. Gene 296, 171–177. doi: 10.1016/S0378-1119(02)00855-7, PMID: 12383514

[ref15] Huang Z. Yan Q. Wang Y. Zou Q. Li J. Liu Z. (2020). Role of mitochondrial dysfunction in the pathology of amyloid-β. J. Alzheimers Dis. 78, 505–514. doi: 10.3233/JAD-200519 33044180

[ref16] Ijaz B. Shabbir A. Shahzad M. Mobashar A. Sharif M. Basheer M. I. (2021). Amelioration of airway inflammation and pulmonary edema by *Teucrium stocksianum* via attenuation of pro-inflammatory cytokines and up-regulation of AQP1 and AQP5. Respir. Physiol. Neurobiol. 284:103569. doi: 10.1016/j.resp.2020.103569, PMID: 33144273

[ref17] Ismail R. Parbo P. Madsen L. S. Hansen A. K. Hansen K. V. Schaldemose J. L. (2020). The relationships between neuroinflammation, beta-amyloid and tau deposition in Alzheimer’s disease: a longitudinal PET study. J. Neuroinflammation 17:151. doi: 10.1186/s12974-020-01820-6, PMID: 32375809 PMC7203856

[ref18] Koníčková D. Menšíková K. Tučková L. Hényková E. Strnad M. Friedecký D. (2022). Biomarkers of neurodegenerative diseases: biology, taxonomy, clinical relevance, and current research status. Biomedicine 10:1760. doi: 10.3390/biomedicines10071760, PMID: 35885064 PMC9313182

[ref19] Li Z. Li H. Zhao C. Lv C. Zhong C. Xin W. (2015). Protective effect of Notoginsenoside R1 on an APP/PS1 mouse model of Alzheimer's disease by up-regulating insulin degrading enzyme and inhibiting Aβ accumulation. CNS Neurol. Disord. Drug Targets 14, 360–369. doi: 10.2174/1871527314666150225141521, PMID: 25714973

[ref20] Loyola A. Almouzni G. (2004). Histone chaperones, a supporting role in the limelight. Biochim. Biophys. Acta 1677, 3–11. doi: 10.1016/j.bbaexp.2003.09.012, PMID: 15020040

[ref21] Mangialasche F. Solomon A. Winblad B. Mecocci P. Kivipelto M. (2010). Alzheimer's disease: clinical trials and drug development. Lancet Neurol. 9, 702–716. doi: 10.1016/S1474-4422(10)70119-8 20610346

[ref22] Manning G. Whyte D. B. Martinez R. Hunter T. Sudarsanam S. (2002). The protein kinase complement of the human genome. Science 298, 1912–1934. doi: 10.1126/science.1075762 12471243

[ref23] Marshall K. E. Vadukul D. M. Staras K. Serpell L. C. (2020). Misfolded amyloid-β-42 impairs the endosomal–lysosomal pathway. Cell. Mol. Life Sci. 77, 5031–5043. doi: 10.1007/s00018-020-03464-4, PMID: 32025743 PMC7658065

[ref24] Meffre D. Grenier J. Bernard S. Courtin F. Dudev T. Shackleford G. (2014). Wnt and lithium: a common destiny in the therapy of nervous system pathologies? Cell. Mol. Life Sci. 71, 1123–1148. doi: 10.1007/s00018-013-1378-1, PMID: 23749084 PMC11113114

[ref25] Misawa T. Arima K. Mizusawa H. Satoh J. (2008). Close association of water channel AQP1 with amyloid-beta deposition in Alzheimer disease brains. Acta Neuropathol. 116, 247–260. doi: 10.1007/s00401-008-0387-x, PMID: 18509662 PMC2516196

[ref26] Nusse R. Clevers H. (2017). Wnt/β-catenin signaling, disease, and emerging therapeutic modalities. Cells 169, 985–999. doi: 10.1016/j.cell.2017.05.016, PMID: 28575679

[ref27] Okuwaki M. Kato K. Nagata K. (2010). Functional characterization of human nucleosome assembly protein 1-like proteins as histone chaperones. Genes Cells 15, 13–27. doi: 10.1111/j.1365-2443.2009.01361.x, PMID: 20002496

[ref28] Panza F. Lozupone M. (2022). The challenges of anti-tau therapeutics in Alzheimer disease. Nat. Rev. Neurol. 18, 577–578. doi: 10.1038/s41582-022-00702-0, PMID: 35941199

[ref29] Park Y. J. Luger K. (2006). Structure and function of nucleosome assembly proteins. Biochem. Cell Biol. 84, 549–558. doi: 10.1139/o06-088, PMID: 16936827

[ref30] Park J. Madan M. Chigurupati S. Baek S. H. Cho Y. Mughal M. R. (2021). Neuronal aquaporin 1 inhibits Amyloidogenesis by suppressing the interaction between Beta-Secretase and amyloid precursor protein. J. Gerontol. A Biol. Sci. Med. Sci. 76, 23–31. doi: 10.1093/gerona/glaa068, PMID: 32154567 PMC7756701

[ref31] Pey P. Pearce R. K. Kalaitzakis M. E. Griffin W. S. Gentleman S. M. (2014). Phenotypic profile of alternative activation marker CD163 is different in Alzheimer's and Parkinson's disease. Acta Neuropathol. Commun. 2:21. doi: 10.1186/2051-5960-2-21, PMID: 24528486 PMC3940003

[ref32] Piras I. S. Krate J. Delvaux E. Nolz J. De Both M. D. Mastroeni D. F. (2019). Association of AEBP1 and NRN1 RNA expression with Alzheimer's disease and neurofibrillary tangle density in middle temporal gyrus. Brain Res. 1719, 217–224. doi: 10.1016/j.brainres.2019.06.004, PMID: 31176712

[ref33] Qiu Q. (2022). Neural networks in autosomal dominant Alzheimer's disease: insights from functional magnetic resonance imaging studies. Front. Aging Neurosci. 14:903269. doi: 10.3389/fnagi.2022.903269, PMID: 35928996 PMC9343946

[ref34] Rodriguez P. Munroe D. Prawitt D. Chu L. L. Bric E. Kim J. (1997). Functional characterization of human nucleosome assembly protein-2 (NAP1L4) suggests a role as a histone chaperone. Genomics 44, 253–265. doi: 10.1006/geno.1997.4868, PMID: 9325046

[ref35] Rodriguez P. Pelletier J. Price G. B. Zannis-Hadjopoulos M. (2000). NAP-2: histone chaperone function and phosphorylation state through the cell cycle. J. Mol. Biol. 298, 225–238. doi: 10.1006/jmbi.2000.3674, PMID: 10764593

[ref36] Rougeulle C. Avner P. (1996). Cloning and characterization of a murine brain specific gene Bpx and its human homologue lying within the Xic candidate region. Hum. Mol. Genet. 5, 41–49. doi: 10.1093/hmg/5.1.41, PMID: 8789438

[ref37] Samaey C. Schreurs A. Stroobants S. Balschun D. (2019). Early cognitive and behavioral deficits in mouse models for tauopathy and Alzheimer’s disease. Front. Aging Neurosci. 11:335. doi: 10.3389/fnagi.2019.00335 31866856 PMC6908963

[ref38] Shen H. H. Huang A. M. Hoheisel J. Tsai S. F. (2001). Identification and characterization of a SET/NAP protein encoded by a brain-specific gene, MB20. Genomics 71, 21–33. doi: 10.1006/geno.2000.6397, PMID: 11161794

[ref39] Simon H. U. Mills G. B. Kozlowski M. Hogg D. Branch D. Ishimi Y. (1994). Molecular characterization of hNRP, a cDNA encoding a human nucleosome-assembly-protein-I-related gene product involved in the induction of cell proliferation. Biochem. J. 297, 389–397. doi: 10.1042/bj2970389, PMID: 8297347 PMC1137842

[ref40] Slomski A. (2022). Anti-tau antibody Semorinemab fails to slow Alzheimer disease. JAMA 328:415. doi: 10.1001/jama.2022.12727 35916858

[ref41] Smith R. J. Dean W. Konfortova G. Kelsey G. (2003). Identification of novel imprinted genes in a genome-wide screen for maternal methylation. Genome Res. 13, 558–569. doi: 10.1101/gr.781503, PMID: 12670997 PMC430166

[ref42] Sun F. Jiang F. Zhang N. Li H. Tian W. Liu W. (2020). Upregulation of Prickle2 ameliorates Alzheimer's disease-like pathology in a transgenic mouse model of Alzheimer's disease. Front. Cell Dev. Biol. 8:565020. doi: 10.3389/fcell.2020.565020, PMID: 33015060 PMC7509431

[ref43] Tahami Monfared A. A. Byrnes M. J. White L. A. Zhang Q. (2022). Alzheimer's disease: epidemiology and clinical progression. Neurol. Ther. 11, 553–569. doi: 10.1007/s40120-022-00338-8, PMID: 35286590 PMC9095793

[ref44] Tan M. G. Chua W. T. Esiri M. M. Smith A. D. Vinters H. V. Lai M. K. (2010). Genome wide profiling of altered gene expression in the neocortex of Alzheimer's disease. J. Neurosci. Res. 88, 1157–1169. doi: 10.1002/jnr.22290, PMID: 19937809

[ref45] Therriault J. Zimmer E. R. Benedet A. L. Pascoal T. A. Gauthier S. Rosa-Neto P. (2022). Staging of Alzheimer's disease: past, present, and future perspectives. Trends Mol. Med. 28, 726–741. doi: 10.1016/j.molmed.2022.05.008, PMID: 35717526

[ref46] Topal F. Ertas B. Guler E. Gurbuz F. Ozcan G. S. Aydemir O. (2022). A novel multi-target strategy for Alzheimer's disease treatment via sublingual route: donepezil/memantine/curcumin-loaded nanofibers. Biomater Adv 138:212870. doi: 10.1016/j.bioadv.2022.212870, PMID: 35913251

[ref47] Uwishema O. Mahmoud A. Sun J. Correia I. F. S. Bejjani N. Alwan M. (2022). Is Alzheimer's disease an infectious neurological disease? A review of the literature. Brain Behav. 12:e2728. doi: 10.1002/brb3.2728 35879909 PMC9392514

[ref48] Vandendriessche C. Kapogiannis D. Vandenbroucke R. E. (2022). Biomarker and therapeutic potential of peripheral extracellular vesicles in Alzheimer's disease. Adv. Drug Deliv. Rev. 190:114486. doi: 10.1016/j.addr.2022.114486 35952829 PMC9985115

[ref49] Wang J. Z. Xia Y. Y. Grundke-Iqbal I. Iqbal K. (2013). Abnormal hyperphosphorylation of tau: sites, regulation, and molecular mechanism of neurofibrillary degeneration. J. Alzheimers Dis. 33, S123–S139. doi: 10.3233/JAD-2012-129031 22710920

[ref50] Watanabe T. K. Fujiwara T. Nakamura Y. Hirai Y. Maekawa H. Takahashi E. (1996). Cloning, expression pattern and mapping to Xq of NAP1L3, a gene encoding a peptide homologous to human and yeast nucleosome assembly proteins. Cytogenet. Cell Genet. 74, 281–285. doi: 10.1159/000134435, PMID: 8976385

[ref51] Yao L. Xu X. Xu Y. Li C. Xie F. Guo M. (2022). OGDHL ameliorates cognitive impairment and Alzheimer's disease-like pathology via activating Wnt/β-catenin signaling in Alzheimer's disease mice. Behav. Brain Res. 418:113673. doi: 10.1016/j.bbr.2021.113673, PMID: 34798170

[ref52] Yu B. Zhang J. Li H. Sun X. (2020). Silencing of aquaporin1 activates the Wnt signaling pathway to improve cognitive function in a mouse model of Alzheimer's disease. Gene 755:144904. doi: 10.1016/j.gene.2020.144904, PMID: 32540373

[ref53] Zhang Z. H. Wu Q. Y. Chen C. Zheng R. Chen Y. Liu Q. (2017b). Selenomethionine attenuates the amyloid-β level by Both inhibiting amyloid-β production and modulating autophagy in neuron-2a/AβPPswe cells. J. Alzheimers Dis. 59, 591–602. doi: 10.3233/JAD-170216, PMID: 28671121

[ref54] Zhang Y. Xu C. Nan Y. Nan S. (2020). Microglia-derived extracellular vesicles carrying miR-711 alleviate Neurodegeneration in a murine Alzheimer's disease model by binding to Itpkb. Front. Cell Dev. Biol. 8:566530. doi: 10.3389/fcell.2020.566530, PMID: 33240878 PMC7677138

[ref55] Zhang H. Zhao C. Cao G. Guo L. Zhang S. Liang Y. (2017a). Berberine modulates amyloid-β peptide generation by activating AMP-activated protein kinase. Neuropharmacology 125, 408–417. doi: 10.1016/j.neuropharm.2017.08.013, PMID: 28822725

