# Supplementary material for: Nucleosome assembly protein 1-like 5 alleviates Alzheimer’s disease-like pathological characteristics in a cell model
Source: Front Mol Neurosci. 2022 Dec 8;15:1034766. doi: 10.3389/fnmol.2022.1034766 (PMC9773259; doi:10.3389/fnmol.2022.1034766)
Supplement: Supplementary file 1 [file Table_1.DOCX]

**Table S1**. **Primers used for RT-qPCR in this study**

| Name | Sequence (5’-3’) |
| --- | --- |
| *NAP1L5*-forward | GCCGAGGACGAGGTAATGG |
| *NAP1L5*-reverse | CATTTCACGGAATTGGGCAAG |
| *AQP1*-forward | AGGCTTCAATTACCCACTGGA |
| *AQP1*-forward | CTTTGGGCCAGAGTAGCGAT |
| β-actin-forward | GGCTGTATTCCCCTCCATCG |
| β-actin-reverse | CCAGTTGGTAACAATGCCATGT |
