# Supplementary material for: Nucleosome assembly protein 1-like 5 alleviates Alzheimer’s disease-like pathological characteristics in a cell model
Source: Front Mol Neurosci. 2022 Dec 8;15:1034766. doi: 10.3389/fnmol.2022.1034766 (PMC9773259; doi:10.3389/fnmol.2022.1034766)
Supplement: Supplementary file 2 [file Table_2.DOCX]

**Table S2. Antibodies used in this study.**

| Antibody (clone) | Cat No. | Region specifity | Species recognition | Host | Sourse | Dilution WB |
| --- | --- | --- | --- | --- | --- | --- |
| NAP1L5 | NBP2-81746 | aa 40-90 of NAP1L5 | Human and mouse | Rabbit | Novus, USA | 1:500 |
| β-Tubulin | 66240-1-Ig | Beta Tubulin fusion protein Ag0117 | Human and mouse | Mouse | Proteintech, China | 1:100000 |
| APP | Ab32136 | clone Y188 to APP | Human and mouse | Rabbit | Abcam, USA | 1:2000 |
| sAPPβ | 813401 | poly8134 | Human and rodent | Rabbit | Biolegend, USA | 1:1000 |
| Aβ | 805501 | clone 12F4 | Human and mouse | Mouse | Biolegend, USA | 1:100 |
| BACE1 | ab183612 | monoclonal antibody [EPR19523] to BACE1 | Human and mouse | Rabbit | Abcam, USA | 1:1000 |
| TAU | T9450 | phosphorylation-independent epitope in amino acids 404-441 (human) | Human and mouse | Mouse | Sigma-Aldrich, USA | 1:1000 |
| TAU (phosphor T231) | ab151559 | human Tau (Phospho T231) polypeptide | Human and mouse | Rabbit | Abcam, USA | 1:2000 |
| TAU (phosphor S396) | ab109396 | human Tau (Phospho S396) polypeptide | Human and mouse | Rabbit | Abcam, USA | 1:2000 |
| GSK3B | 22104-1-AP | GSK3B fusion protein Ag17320 | Human and mouse | Rabbit | Proteintech, China | 1:2000 |
| Phospho-GSK3B (Ser9) | 9336 | synthetic phosphopeptides around S9 of human GSK3B | Human and mouse | Rabbit | CST, USA | 1:1000 |
| β-Catenin | sc7963 | mouse monoclonal antibody raised against amino acids  680-781 mapping at the C-terminus of β-catenin of human origin. | Human and mouse | Mouse | Santa Cruz, Argentina | 1:1000 |
| Phospho-β-Catenin (Ser33/37/Thr41) | 9561 | synthetic phosphopeptides corresponding to residues around Ser33, Ser37, and Thr41 of human β-Catenin | Human and mouse | Rabbit | CST, USA | 1:1000 |
| AQP1 | WLH3886 | Polyclonal antibody is produced by immunizing animals with a synthetic  peptide of AQP1 | Human and mouse | Rabbit | Wanleibio, China | 1:1000 |
