# Supplementary material for: Nucleosome assembly protein 1-like 5 alleviates Alzheimer’s disease-like pathological characteristics in a cell model
Source: Front Mol Neurosci. 2022 Dec 8;15:1034766. doi: 10.3389/fnmol.2022.1034766 (PMC9773259; doi:10.3389/fnmol.2022.1034766)
Supplement: Supplementary file 3 [file Table_3.DOCX]

**Table S3. Antibodies used for immunofluorescence in this study.**

| Antibody (clone) | Cat No. | Region specifity | Species recognition | Host | Sourse | Dilution |
| --- | --- | --- | --- | --- | --- | --- |
| NAP1L5 | bs-19021R | KLH conjugated synthetic peptide derived from human NAP1L5: 51-132/132 | Human and mouse | Rabbit | Bioss, China | 1:100 |
| α-SMA | 67735-1-Ig | Peptide of α-SMA | Human and mouse | Mouse | Proteintech, China | 1:200 |
| NeuN | 66836-1-Ig | NeuN fusion protein Ag28016 | Human and mouse | Mouse | Proteintech, China | 1:100 |
