# Supplementary material for: Nucleosome assembly protein 1-like 5 alleviates Alzheimer’s disease-like pathological characteristics in a cell model
Source: Front Mol Neurosci. 2022 Dec 8;15:1034766. doi: 10.3389/fnmol.2022.1034766 (PMC9773259; doi:10.3389/fnmol.2022.1034766)
Supplement: Supplementary file 4 [file Data_Sheet_1.PDF]

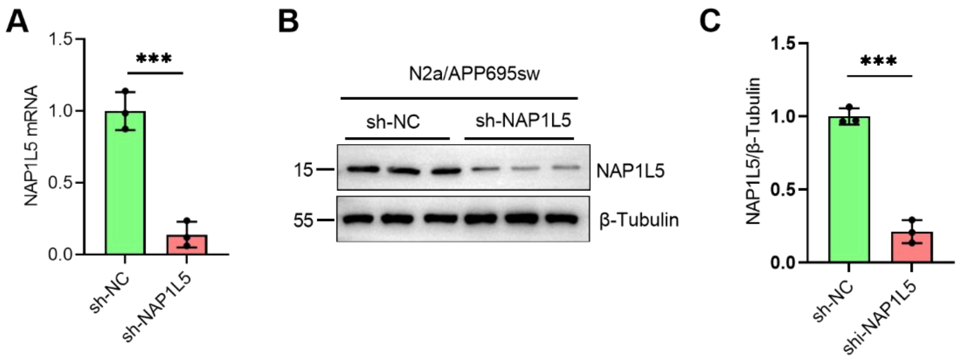

Fig. S1 RT-qPCR and Western bolt detected the mRNA and protein levels of NAP1L5. N2a-APP695sw cells were infected with lentivirus sh-NC or sh-NAP1L5 vectors, then the stable cell line was obtained by addition of puromycin (4 μg/ml final concentration). (A) RT-qPCR was used to detect the mRNA level in NAP1L5 knockdown cells. (B) Western blot was used to detect the NAP1L5 protein level in NAP1L5 knockdown cells. Data are present as means ± SD, n = 3. $^{***}p <$ 0.001; Student's test.
